# Clinical role and biological function of CDK5 in hepatocellular carcinoma: A study based on immunohistochemistry, RNA-seq and *in vitro* investigation

**DOI:** 10.18632/oncotarget.22659

**Published:** 2017-11-26

**Authors:** Rui Zhang, Peng Lin, Hong Yang, Yun He, Yi-Wu Dang, Zhen-Bo Feng, Gang Chen

**Affiliations:** ^1^ Department of Pathology, First Affiliated Hospital of Guangxi Medical University, Nanning, Guangxi Zhuang Autonomous Region, P. R. China; ^2^ Department of Ultrasonography, First Affiliated Hospital of Guangxi Medical University, Nanning, Guangxi Zhuang Autonomous Region, P. R. China

**Keywords:** CDK5, hepatocellular carcinoma, immunohistochemistry, The Cancer Genome Atlas, siRNA, Pathology Section

## Abstract

To investigate the clinical role and biological function of cyclin-dependent kinase 5 (CDK5) in hepatocellular carcinoma (HCC), 412 surgically resected tissue samples (HCC, n=171; non-HCC=241) were obtained and analyzed with immunohistochemistry. The diagnostic and prognostic values of CDK5 expression levels in HCC were clarified. Moreover, RNA-seq data or microarray datasets from The Cancer Genome Atlas (TCGA) (HCC, n=374; normal, n=50) or other public databases (HCC, n=1864; non-tumor=1995) regarding CDK5 in HCC were extracted and examined. Several bioinformatic methods were performed to identify CDK5-regulated pathways. *In vitro* experiments were adopted to measure proliferation and apoptosis in HCC cells after CDK5 mRNA was inhibited in the HCC cell lines HepG2 and HepB3. Based on immunohistochemistry, CDK5 expression levels were notably increased in HCC tissues (n=171) compared with normal (n=33, *P*<0.001), cirrhosis (n=37, *P*<0.001), and adjacent non-cancerous liver (n=171, *P*<0.001) tissues. The up-regulation of CDK5 was associated with higher differentiation (*P*<0.001), metastasis (*P*<0.001), advanced clinical TNM stages (*P*<0.001), portal vein tumor embolus (*P*=0.003) and vascular invasion (*P*=0.004). Additionally, TCGA data analysis also revealed significantly increased CDK5 expression in HCC compared with non-cancerous hepatic tissues (*P*<0.001). The pooled standard mean deviation (SMD) based on 36 included datasets (HCC, n=2238; non-cancerous, n=2045) indicated that CDK5 was up-regulated in HCC (SMD=1.23, 95% CI: 1.00-1.45, *P*<0.001). The area under the curve (AUC) of the summary receiver operating characteristic (SROC) curve was 0.88. Furthermore, CDK5 knock-down inhibited proliferation and promoted apoptosis. In conclusion, CDK5 plays an essential role in the initiation and progression of HCC, most likely via accelerating proliferation and suppressing apoptosis in HCC cells by regulating the cell cycle and DNA replication pathways.

## INTRODUCTION

Ranked as the fifth common type of cancer worldwide, hepatocellular carcinoma (HCC) ranks as the third cause of cancer-related deaths [1]. In particular, given the wide spread of hepatic virus, people in developing country are more susceptible to HCC [2]. HCC is characterized by its early invasion and diffuse metastases characteristics [3]. Depressingly, the lack of ideal biomarkers consistently leads to HCC diagnostic delay. For example, alpha-fetoprotein (AFP) assessment lacks adequate sensitivity and specificity for diagnosis [1, 4]. Thus, a majority of patients suffering from HCC are unable to obtain a definite diagnosis until advanced stage disease, making HCC one of the most frequent cancers worldwide [5]. Moreover, given its characteristics of toxicity and resistance to chemotherapy and radiotherapy, the prognosis of HCC remains poor to date [6-8]. The mortality rate of HCC is increasing despite significant progress in diagnosis and treatment obtained over the last few years. However, the 5-year survival rate of HCC is only 5% [9]. Therefore, the identification of a target gene strongly associated with HCC is of great value for HCC prevention and diagnosis.

As one of the members of the CDK family, CDK5 acts as an important regulator of cell division cycle and was first discovered and reported in 1992 [10, 11]. In addition to its role in brain tissues, CDK5 plays a key role in various types of cancer, including gastric cancer, prostate cancer, and lung cancer [12-15]. Recently, several publications also reported high CDK5 expression levels in hepatocellular carcinoma [15, 16]. As previously reported, CDK5 is highly expressed in HCC tissues and regulates the DNA damage response to influence its downstream cascade [15]. Herzog J et al. demonstrated that CDK5 promotes angiogenesis in hepatocellular carcinoma by its interaction with the transcription factor HIF-1α [16]. However, the sample size of the study was small. Only 157 HCC samples were included in the study by Ehrlich SM et al. More are needed to support the finding. Moreover, the relationship between CDK5 and the clinical variables of HCC remain unclear. Thus, using immunohistochemistry (IHC) in combination with high-throughput RNA-sequencing (RNA-seq) or microarray data from The Cancer Genome Atlas (TCGA), Gene Expression Omnibus (GEO), ArrayExpress and Oncomine databases, our study seeks to confirm the relationship among CDK5 expression levels and HCC development and progression. Subsequently, the role of CDK5 in cell cycle pathways was discovered using bioinformatics methods. Given that siRNA is widely used to interfere with gene expression, we used CDK5 siRNA to transfect HCC cells *in vitro* and assessed HCC cell proliferation and apoptosis.

## RESULTS

### Differential CDK5 protein expression from our institution and from Protein Atlas detected by immunohistochemistry

**Figure 1:**
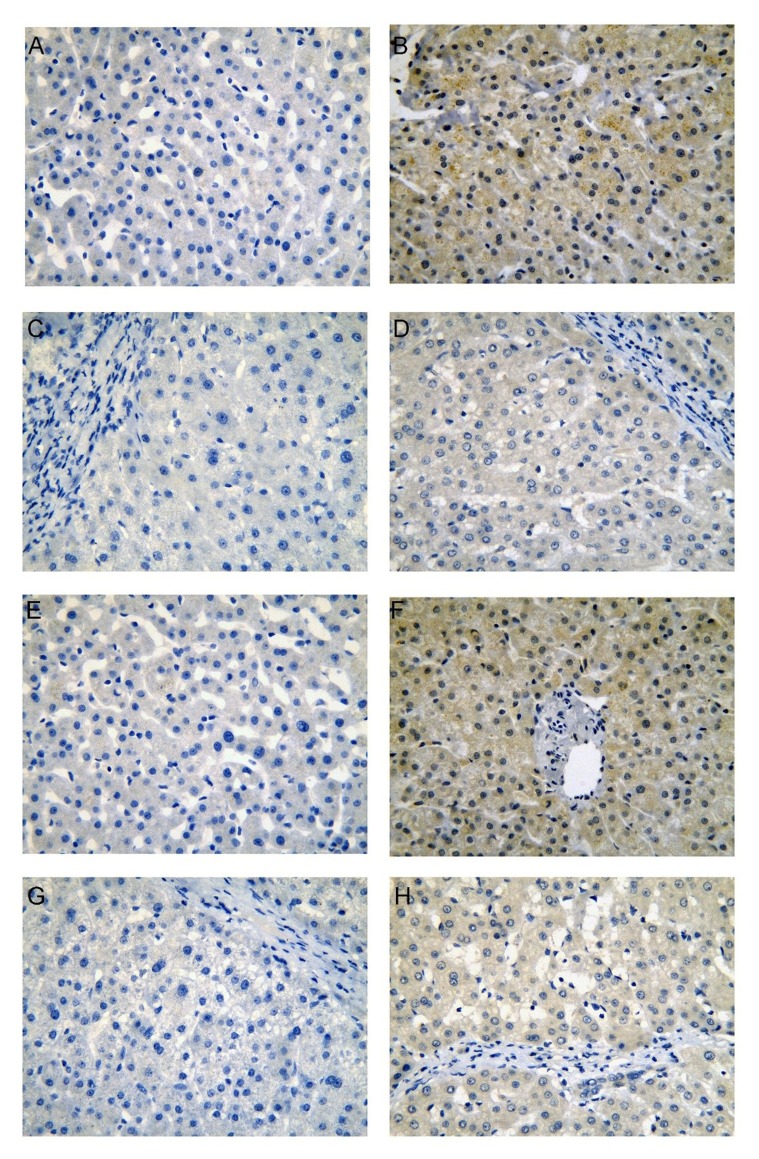
CDK5 protein expression in non-HCC liver tissues from our institution Normal liver (**A**, negative; **B**, positive), cirrhotic liver (**C**, negative; **D**, positive), para-tumorous normal liver (**E**, negative; **F**, positive), para-tumorous cirrhotic liver (**G**, negative; **H**, positive), immunohistochemistry, ×400.

**Figure 2:**
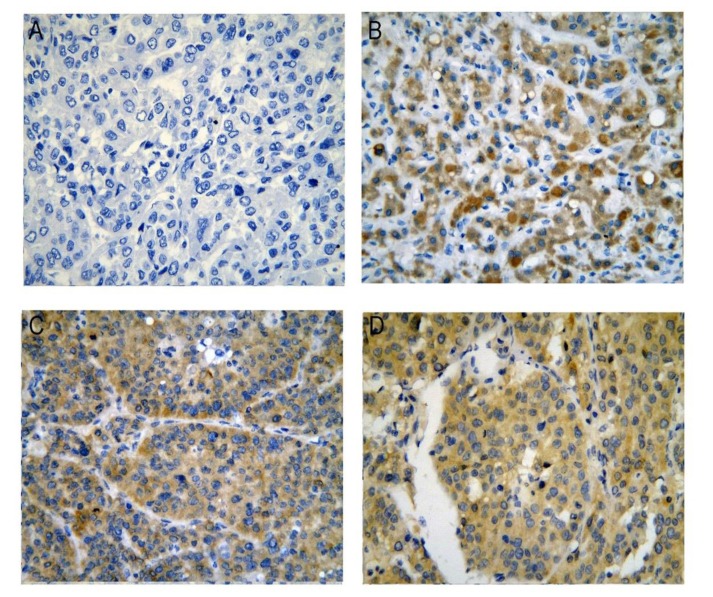
CDK5 protein expression in HCC tissues from our institution (**A**) Negative; (**B**), (**C**), (**D**) Positive, immunohistochemistry, ×400.

**Figure 3:**
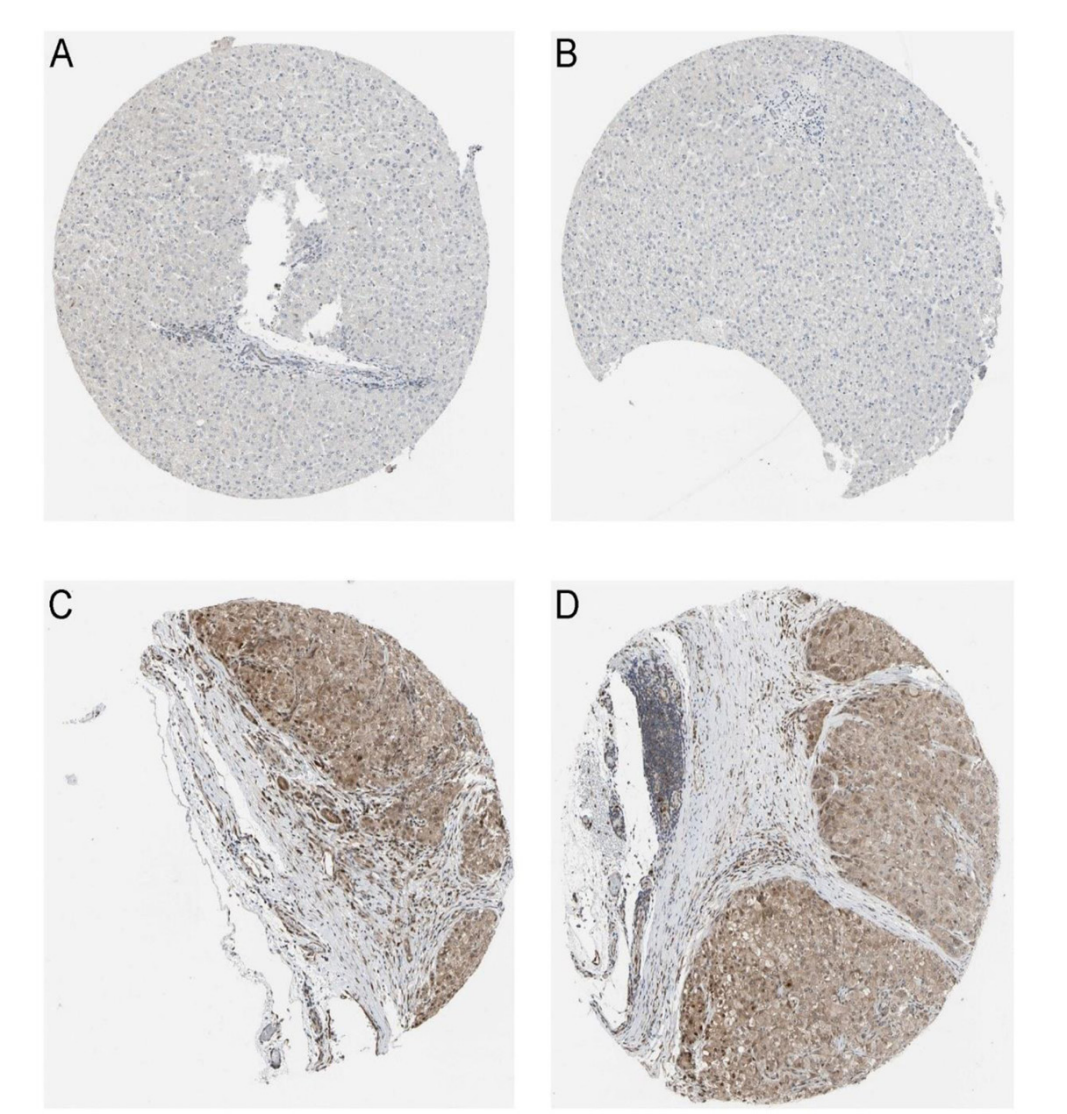
CDK5 protein in normal liver and HCC tissues from Protein Atlas (**A**, **B**), Normal liver tissues stain negative for CDK5, immunohistochemistry, ×100; (**C**, **D**), HCC tissues stain positive for CDK5, immunohistochemistry, ×100.

An increasing tendency for CDK5 positive rates was observed from normal liver tissues (n=33), cirrhotic tissues (n=37), adjacent non-HCC liver tissues (n=171) to HCC tissues (n=171) (χ2=53.450, *P*<0.001) (Table 1, Figure 1, Figure 2). Additionally, the area under the curve (AUC) of receiver operator characteristic curves (ROC) was 0.678 (95% CI: 0.625-0.730, *P*<0.001) for CDK5 protein to diagnose HCC, which indicated a certain value for clinical diagnosis of HCC. HCC patients with metastasis (n=81), portal vein tumor (n=45), vascular invasion (n=52) and advanced TNM stage (n=123) exhibited prominently increased CDK5 expression (*P*<0.01) (Table 1). Moreover, remarkable overexpression of CDK5 protein was confirmed by the independent cases from Protein Atlas, which revealed the absence of CDK5 in normal livers and moderate-strong CDK5 staining in HCC (Figure 3).

**Table 1:** Relationship between CDK5 levels and clinicopathological variables in HCC from our institution

Variables		n	Expression of CDK5 (%)		*χ*^2^ value	*P* value
			Negative	Positive		
Tissue types	Normal liver	33	23 (69.7)	10 (30.3)	53.450	<0.001
	Cirrhosis	37	23 (62.2)	14 (37.8)		
	Adjacent non-cancerous liver	171	96 (56.1)	75 (43.9)		
	HCC	171	40 (23.4)	131 (76.6)		
Gender	Male	153	35 (22.9)	118 (77.1)	0.216	0.768
	Female	18	5 (27.8)	13 (72.2)		
Differentiation	High	20	12 (60.0)	8 (40.0)	17.161	<0.001
	Moderate	98	17 (17.3)	81 (82.7)		
	Low	53	11 (20.8)	42 (79.2)		
Size	<5 cm	58	19 (32.8)	39 (67.2)	4.297	0.055
	≥5 cm	113	21 (18.6)	92 (81.4)		
Tumor nodes	Single	68	13 (19.1)	55 (80.9)	0.163	0.819
	Multiple	61	10 (16.4)	51 (83.6)		
Metastasis	-	90	38 (42.2)	52 (57.8)	37.595	<0.001
	+	81	2 (2.5)	79 (97.5)		
Clinical TNM stage	I-II	48	22 (45.8)	26 (54.2)	18.754	<0.001
	III-IV	123	18 (14.6)	105 (85.4)		
Portal vein tumor embolus	-	84	21 (25.0)	63 (75.0)	8.451	0.003
	+	45	2 (4.4)	43 (95.6)		
Vaso-invasion	-	77	20 (26.0)	57 (74.0)	8.649	0.004
	+	52	3 (5.8)	49 (94.2)		
Tumor capsular infiltration	With complete capsule	61	12 (19.7)	49 (80.3)	0.268	0.650
	Infiltration or no capsule	68	11 (16.2)	57 (83.8)		
AFP	-	56	12 (21.4)	44 (78.6)	0.146	0.813
	+	54	10 (18.5)	44 (81.5)		
Cirrhosis	-	74	13 (17.6)	61 (82.4)	2.469	0.145
	+	97	27 (27.8)	70 (72.2)		

### Verification of CDK5 mRNA expression based on TCGA data

**Figure 4:**
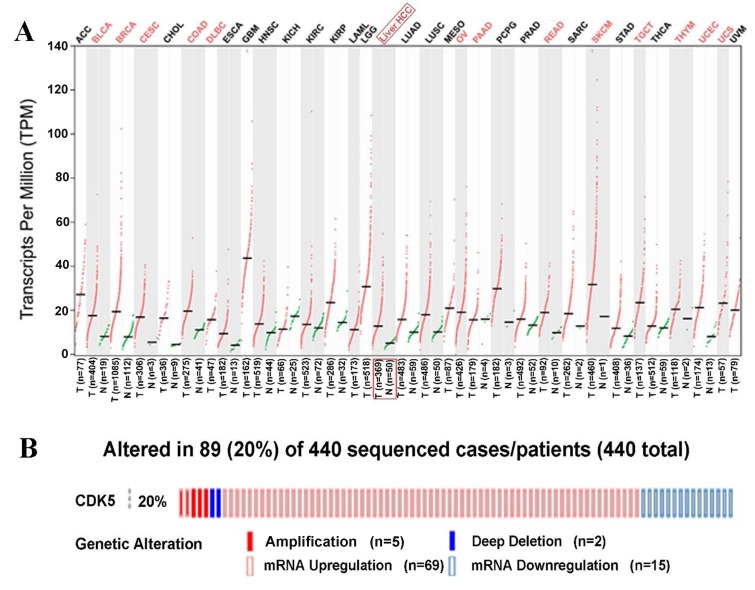
CDK5 expression pattern from The Cancer Genome Atlas and genetic alteration from cBioPortal (**A**) Transcripts Per Million (TPM) data of CDK5 expression are presented based on Gene Expression Profiling Interactive Analysis (GEPIA). (**B**) Genetic alteration of CDK5 in 440 HCC patients from cBioPortal. CDK5 was altered in a total of 89 HCC patients. CDK5 amplificated in 5 patients and deep deleted in 2 patients. Meanwhile, CDK5 upregulated in 69 cases but downregulated in 15 cases.

**Figure 5:**
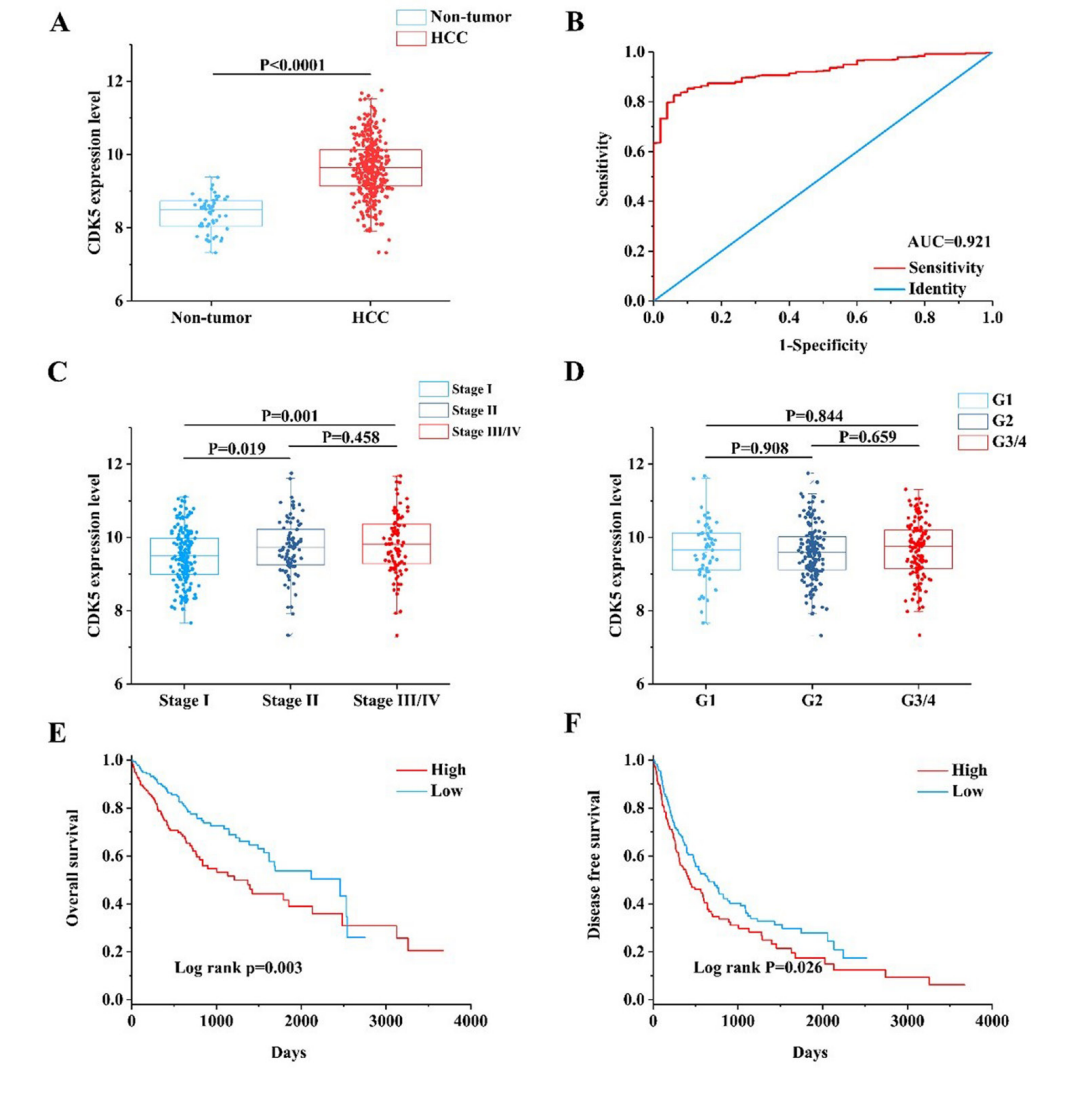
Clinical value of CDK5 in HCC based on TCGA data (**A**) Scatter plot of CDK5 expression in HCC and cancer-free normal liver tissues. (**B**) Receiver operating characteristic (ROC) curve of CDK5 in HCC. (**C**) Scatter plot of CDK5 expression at different pathological stages. (**D**) Scatter plot of CDK5 expression at different histological grades. (**E**) Kaplan-Meier plots revealed an association between increased CDK5 levels and reduced overall survival. (**F**) Kaplan-Meier plots revealed an association between increased CDK5 levels and reduced disease-free survival.

First, we observed the CDK5 expression pattern in 33 types of tumors based on TCGA data. CDK5 was significantly increased in 14 cancers, including liver HCC (Figure 4A). In total, 374 HCC patients and 50 patients without hepatic cancer from the TCGA database were included in this study. CDK5 expression levels were increased in HCC tissues compared with paired normal liver tissues (9.6443±0.7757 vs. 8.3711±0.4678, *P*<0.0001) (Figure 5A, Table 2). The ROC curve was performed to evaluate the significance of CDK5 expression in the diagnosis of HCC, and the area under curve (AUC) was 0.921 (Figure 5B). CDK5 expression increased in patients older than 60 years (n=201) compared with patients less than 60 years of age (n=169) (9.7650±0.7477 vs. 9.4965±0.7752, *P*<0.001), increased in males (n=250) compared with females (n=121) (9.7079±0.7568 vs. 9.5024±0.7833, *P*=0.016), increased in pathologic stages III-IV (n=90) compared with pathologic stages I-II (n=257) (9.8117±0.8200 vs. 9.5675±0.7513, *P*=0.010), and increased in T3-T4 stage (n=93) compared with T1-T2 stage (n=275) (9.8115±0.7956 vs. 9.5905±0.7491, *P*=0.016). Nevertheless, there are no significant differences between CDK5 expression level and other related pathological subgroups, such as race, relative family cancer history, tumor status, histological grade, N stage, M stage, and vascular tumor cell type (Table 2). We also generated plots to provide a visual representation of CDK5 expression in different pathological stages and histological grades (Figure 5C, Figure 5D).

**Table 2:** Relationship between CDK5 level and clinicopathological parameters in HCC based on TCGA data

Parameters		n	Mean value	t value	*P* value
Tissues	HCC	374	9.6443±0.7757	16.457	<0.001
	Normal	50	8.3711±0.4678		
Age	≥60	201	9.7650±0.7477	3.383	0.001
	<60	169	9.4965±0.7752		
Gender	Male	250	9.7079±0.7568	2.424	0.016
	Female	121	9.5024±0.7833		
Race	White	184	9.7074±0.7072	1.921	0.056
	Asian	158	9.5428±0.8551		
Relative family cancer history	Yes	112	9.7301±0.7253	1.754	0.080
	No	208	9.5709±0.7993		
Tumor status	With tumor	151	9.6705±0.7830	0.859	0.391
	Tumor free	201	9.5985±0.7733		
Histological grade	G3∼G4	134	9.6562±0.7759	0.423	0.672
	G1∼G2	232	9.6208±0.7692		
Pathologic stage	III∼IV	90	9.8117±0.8200	2.591	0.010
	I∼II	257	9.5675±0.7513		
T stage	T3-T4	93	9.8115±0.7956	2.42	0.016
	T1-T2	275	9.5905±0.7491		
N stage	N1-3	4	9.9694±1.0897	0.842	0.401
	N0	252	9.6372±0.7787		
M stage	M1	4	9.3233±0.2386	-0.807	0.420
	M0	266	9.6451±0.7953		
Vascular tumor cell type	Micro/Macro	109	9.6338±0.7354	0.326	0.745
	None	205	9.6045±0.7681		

### Examination of the CDK5 expression pattern in HCC based on other open databases

**Figure 6:**
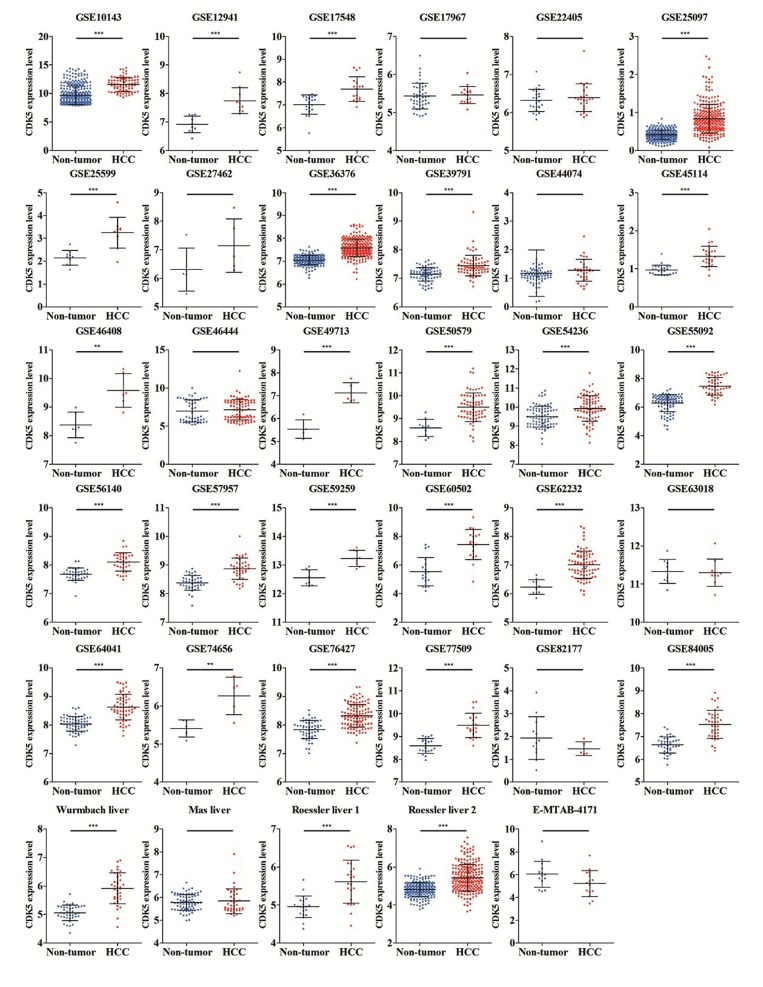
Different levels of CDK5 expression in HCC and non-tumor gastric tissues based on 35 datasets

**Figure 7:**
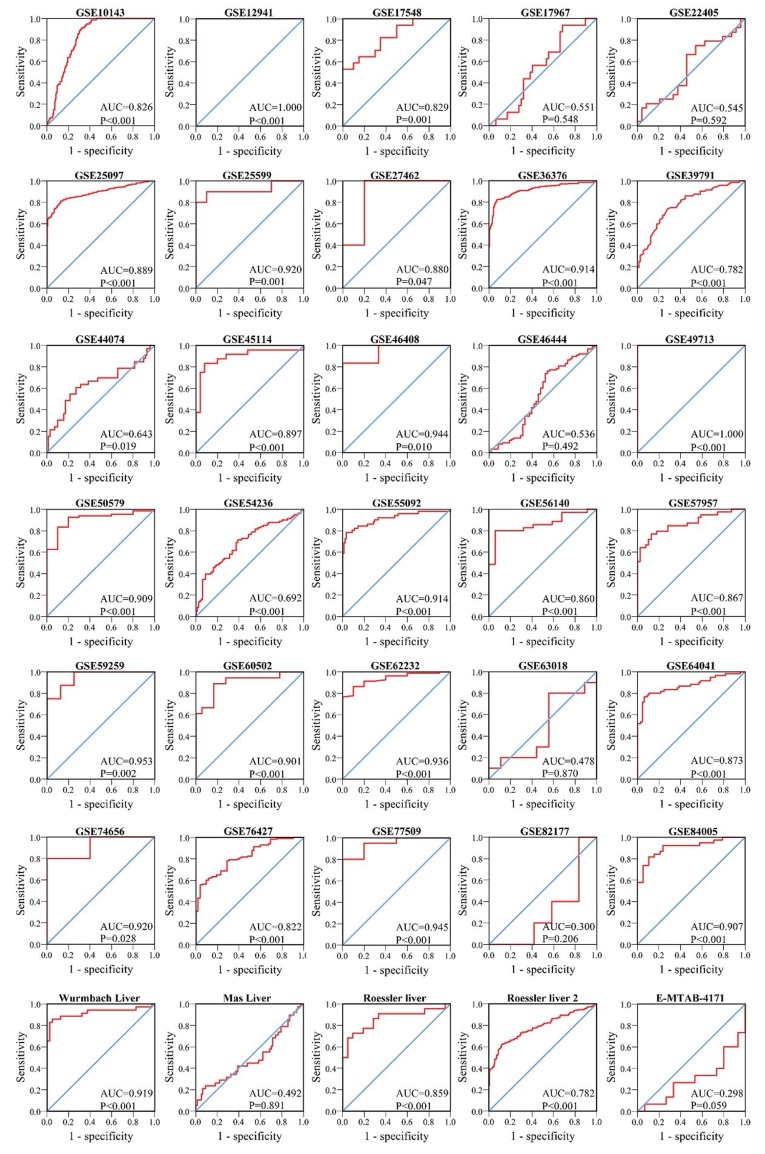
ROC curves of CDK5 expression for the differentiation of HCC from non-tumor tissues based on 35 datasets

**Figure 8:**
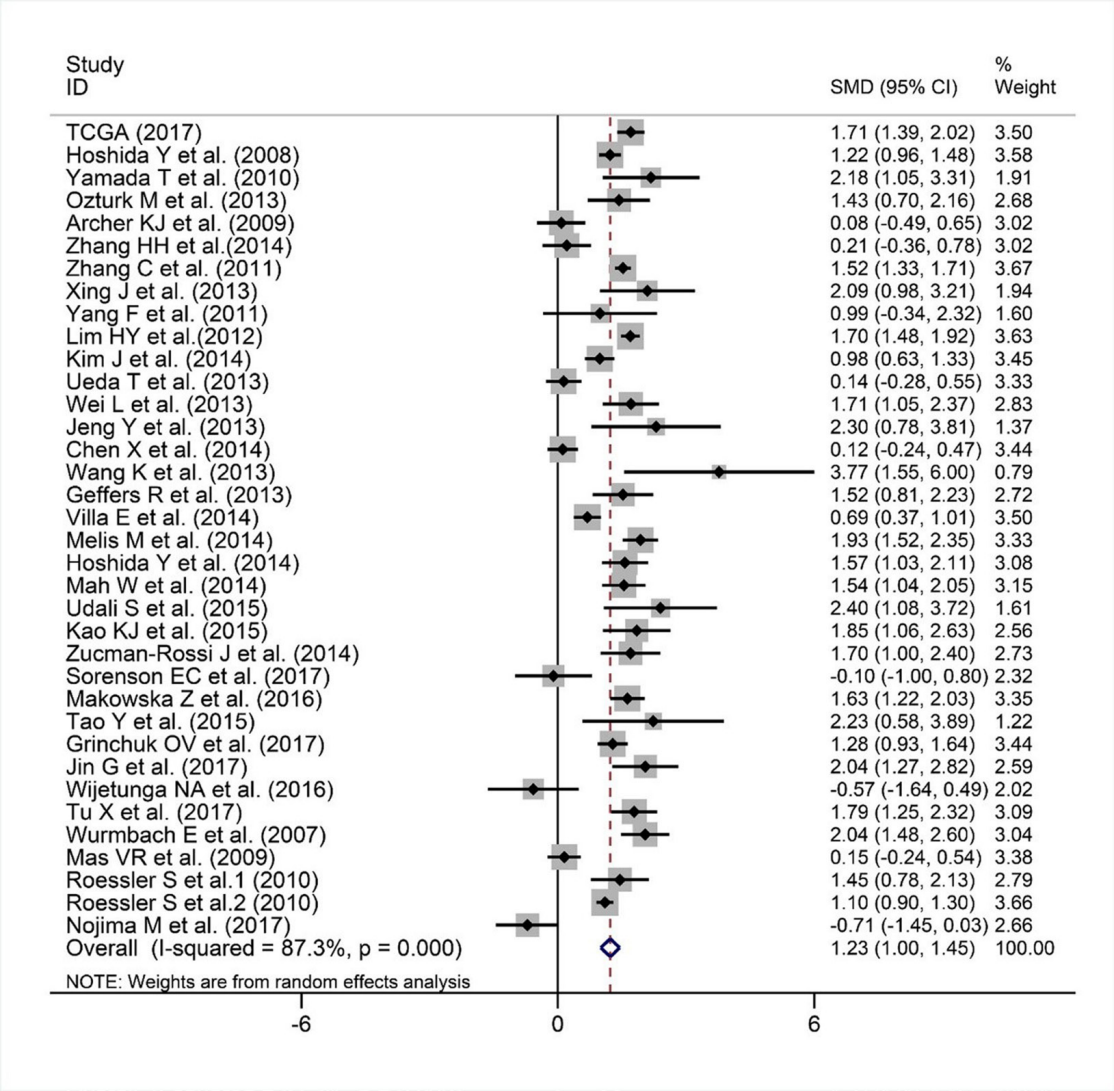
Forest plot evaluating CDK5 expression between HCC and non-tumor tissues When SMD > 0 and its 95% CI do not cross, 0 indicates increased CDK5 expression in HCC compared with noncancerous samples.

**Figure 9:**
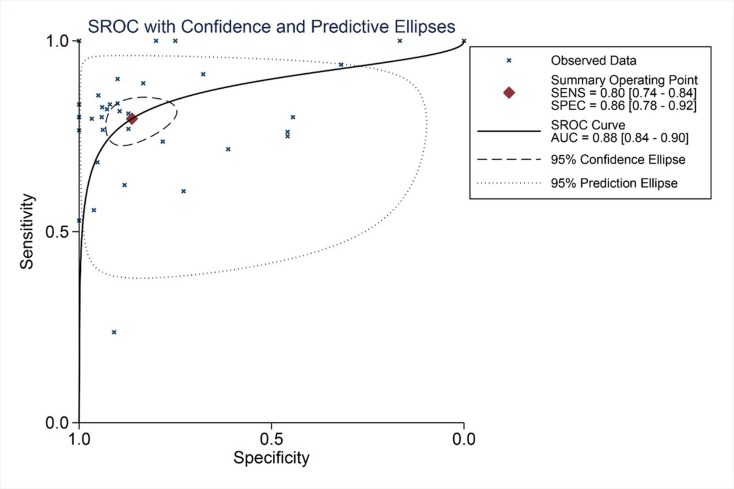
SROC curves for the differentiation of HCC patients from non-tumor tissues based on CDK5 expression

We finally obtained 35 RNA-seq or microarray datasets, which provided CDK5 expression value in HCC tissues (n=1864) and adjacent non-tumor tissues (n=1995), from online databases (GEO, ArrayExpress and Oncomine databases). All included datasets are summarized in Table 3. CDK5 expression was significantly increased in HCC tissues (n=1630) compared with non-tumor tissues (n=1688) based on 26 of these datasets. In addition, CDK5 expression did not differ between HCC tissues (n=234) and non-tumor tissues (n=307) in the other 9 datasets. Scatter plots and ROC curve plots were drawn to visually represent the results (Figure 6, Figure 7). A comprehensive integrated approach was deemed to be more credible than single-dataset analysis. The pooled SMD reached 1.23 (95% CI: 1.00-1.45, *P*<0.001) by the random-effects model (Figure 8), certifying that CDK5 is overexpressed in HCC. Furthermore, the meta-analysis results for testing the diagnostic value of CDK5 revealed that the AUC of SROC was 0.88 (95% CI: 0.84-0.90) (Figure 9) Interestingly, as shown in Figure 4B, CDK5 also has a higher percentage (77.52%, n=69) in mRNA upregulation in genetic alteration from cBioPortal.

**Table 3:** Characteristics of datasets collected from public databases

First author (publication year)	Country	Dataset	Platform	Cancer			Non-tumor		
				N	Mean	SD	N	Mean	SD
Hoshida Y et al. (2008)	USA	GEO: GSE10143	Illumina GPL5474	80	11.56476	1.234071	307	9.681402	1.612077
Yamada T et al. (2010)	Japan	GEO: GSE12941	Affymetrix GPL5175	10	7.742833	0.451358	10	6.916281	0.289366
Ozturk M et al. (2013)	Turkey	GEO: GSE17548	Affymetrix GPL570	17	7.689396	0.534538	20	7.010264	0.419098
Archer KJ et al. (2009)	USA	GEO: GSE17967	Affymetrix GPL571	16	5.457902	0.224498	47	5.432252	0.335892
Zhang HH et al. (2014)	USA	GEO: GSE22405	Affymetrix GPL10553	24	6.385462	0.363429	24	6.316088	0.292544
Zhang C et al. (2011)	USA	GEO: GSE25097	Rosetta GPL10687	268	0.838214	0.378756	289	0.416037	0.122694
Xing J et al. (2013)	China	GEO: GSE25599	Illumina GPL9052	10	3.244943	0.671844	10	2.143752	0.319294
Yang F et al. (2011)	China	GEO: GSE27462	Arraystar GPL11269	5	7.140901	0.933327	5	6.30459	0.751626
Lim HY et al.(2012)	South Korea	GEO: GSE36376	Illumina GPL10558	240	7.574646	0.378203	193	7.045063	0.201359
Kim J et al. (2014)	USA	GEO: GSE39791	Illumina GPL10558	72	7.443333	0.361277	72	7.144306	0.235347
Ueda T et al. (2013)	Japan	GEO: GSE44074	Kanazawa GPL13536	33	1.27919	0.383991	70	1.182476	0.812676
Wei L et al. (2013)	China	GEO: GSE45114	CapitalBio GPL5918	24	1.325073	0.268345	25	0.967549	0.128934
Jeng Y et al. (2013)	Taiwan	GEO: GSE46408	Agilent GPL4133	6	9.579482	0.587683	6	8.377478	0.449755
Chen X et al. (2014)	USA	GEO: GSE46444	Illumina GPL13369	88	7.143259	1.327809	48	6.98491	1.454663
Wang K et al. (2013)	China	GEO: GSE49713	Arraystar GPL11269	5	7.124351	0.441245	5	5.535282	0.400369
Geffers R et al. (2013)	Germany	GEO: GSE50579	Agilent GPL14550	67	9.499062	0.624212	10	8.586041	0.373536
Villa E et al. (2014)	Italy	GEO: GSE54236	Agilent GPL6480	81	9.92021	0.665807	80	9.4993	0.546339
Melis M et al. (2014)	USA	GEO: GSE55092	Affymetrix GPL570	49	7.458387	0.616179	91	6.286526	0.60129
Hoshida Y et al. (2014)	USA	GEO: GSE56140	Illumina GPL18461	35	8.10847	0.32	34	7.678189	0.217079
Mah W et al. (2014)	Singapore	GEO: GSE57957	Illumina GPL10558	39	8.869112	0.369617	39	8.37716	0.257911
Udali S et al. (2015)	Italy	GEO: GSE59259	NimbleGen GPL18451	8	13.22883	0.281264	8	12.55599	0.279291
Kao KJ et al. (2015)	Taiwan	GEO: GSE60502	Affymetrix GPL96	18	7.425576	1.055023	18	5.531417	0.995259
Zucman-Rossi J et al. (2014)	France	GEO: GSE62232	Affymetrix GPL570	81	7.008307	0.476591	10	6.227086	0.257979
Sorenson EC et al. (2017)	USA	GEO: GSE63018	Illumina GPL16791	10	11.29778	0.353735	9	11.33185	0.316222
Makowska Z et al. (2016)	Switzerland	GEO: GSE64041	Affymetrix GPL6244	60	8.627706	0.445288	65	8.042826	0.255531
Tao Y et al. (2015)	China	GEO: GSE74656	Affymetrix GPL16043	5	6.26234	0.491062	5	5.410328	0.223788
Grinchuk OV et al. (2017)	Singapore	GEO: GSE76427	Illumina GPL10558	115	8.325151	0.398464	52	7.843747	0.317144
Jin G et al. (2017)	China	GEO: GSE77509	Illumina GPL16791	20	9.487431	0.532998	20	8.588088	0.320587
Wijetunga NA et al. (2016)	USA	GEO: GSE82177	Illumina GPL11154	5	1.467352	0.301874	12	1.933381	0.931835
Tu X et al. (2017)	China	GEO: GSE84005	Affymetrix GPL5175	38	7.528008	0.610855	38	6.635592	0.355587
Wurmbach E et al. (2007)	USA	Oncomine: Wurmbach Liver	Affymetrix GPL570	35	5.919037	0.545655	40	5.059372	0.269329
Mas VR et al. (2009)	USA	Oncomine: Mas Liver	Affymetrix GPL571	38	5.842322	0.548437	77	5.777008	0.347634
Roessler S et al.1 (2010)	USA	Oncomine: Roessler liver 1	Affymetrix GPL571	22	5.611227	0.566651	21	4.954476	0.287342
Roessler S et al.2 (2010)	USA	Oncomine: Roessler liver 2	Affymetrix GPL3921	225	5.4402	0.703646	220	4.819882	0.365017
Nojima M et al. (2017)	Japan	Arrayexpress: E-MTAB-4171	Agilent A-MEXP-2320	15	5.237674	1.130954	15	6.042401	1.132882

### Impact of CDK5 expression on survival outcomes in hepatic cancer

Kaplan-Meier plots were adopted to analyze the survival differences between low and high CDK5 expression levels with the cutoff value defined by the median CDK5 expression level (Figure 5E, Figure 5F). The plots indicated that the HCC patients with a high expression of CDK5 had an inferior overall survival (OS; HR=1.697, 95% CI: 1.195-2.410, P=0.003) and disease-free survival (DFS; HR=1.351, 95% CI: 1.036-1.763, P=0.026) than those patients with a downregulated expression of CDK5.

### Bioinformatic analysis suggests that CDK5 is associated with the proliferative signaling pathway

**Figure 10:**
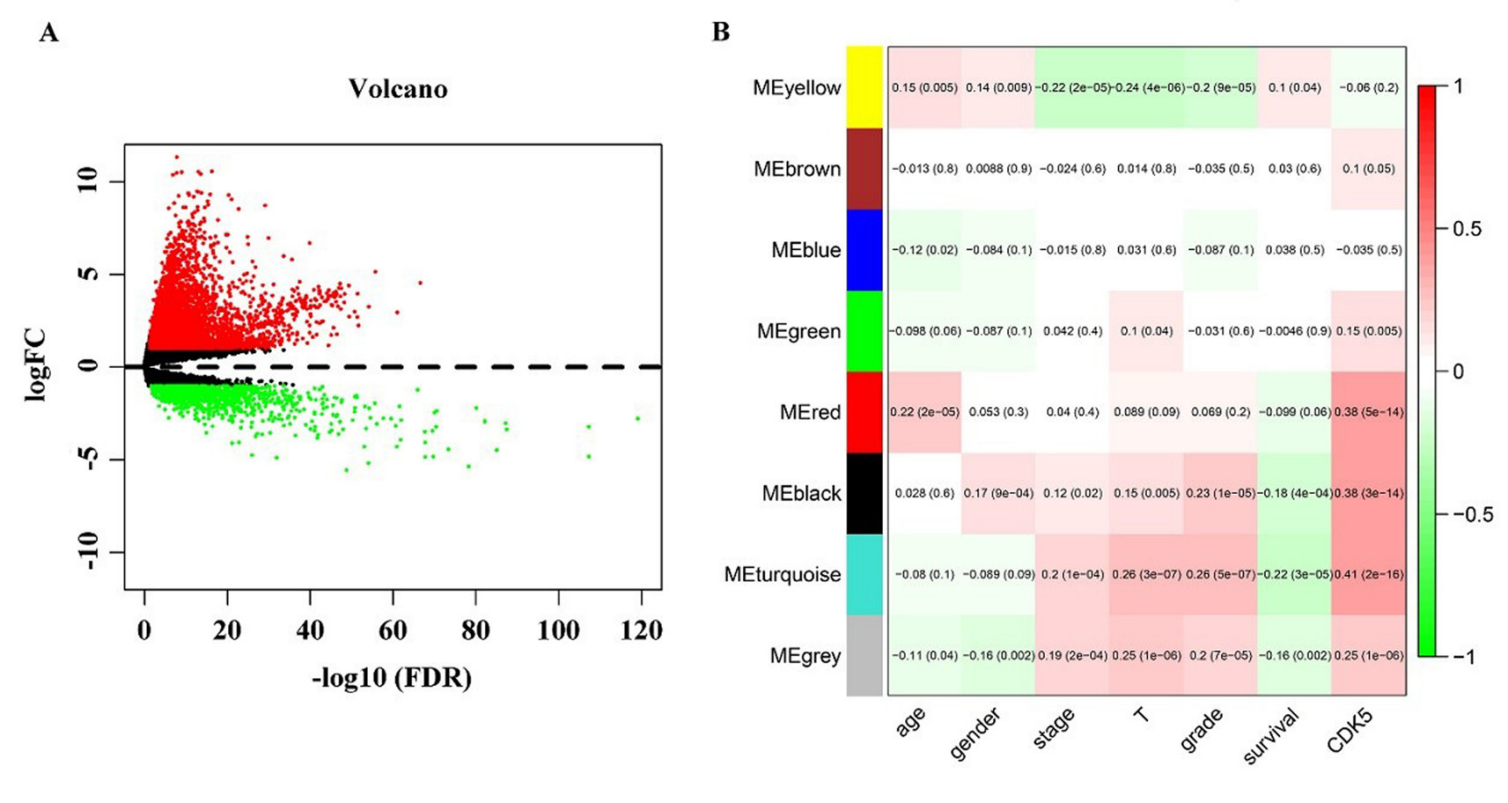
Identification of CDK5-related genes (**A**) Volcano plot of the differentially expressed genes between liver HCC and normal liver tissues. Red indicates high expression, whereas green represents low expression. This volcano plot was generated using the ggplot2 package of R language. (**B**) Network analysis of differently expressed genes identifies a module of genes co-expressed with CDK5. Each row corresponds to a module eigengene, and each column corresponds to a clinicopathological parameter. Each block contains the corresponding correlation coefficient and *P* value. The heatmap was drawn using the WGCNA package of R language.

**Figure 11:**
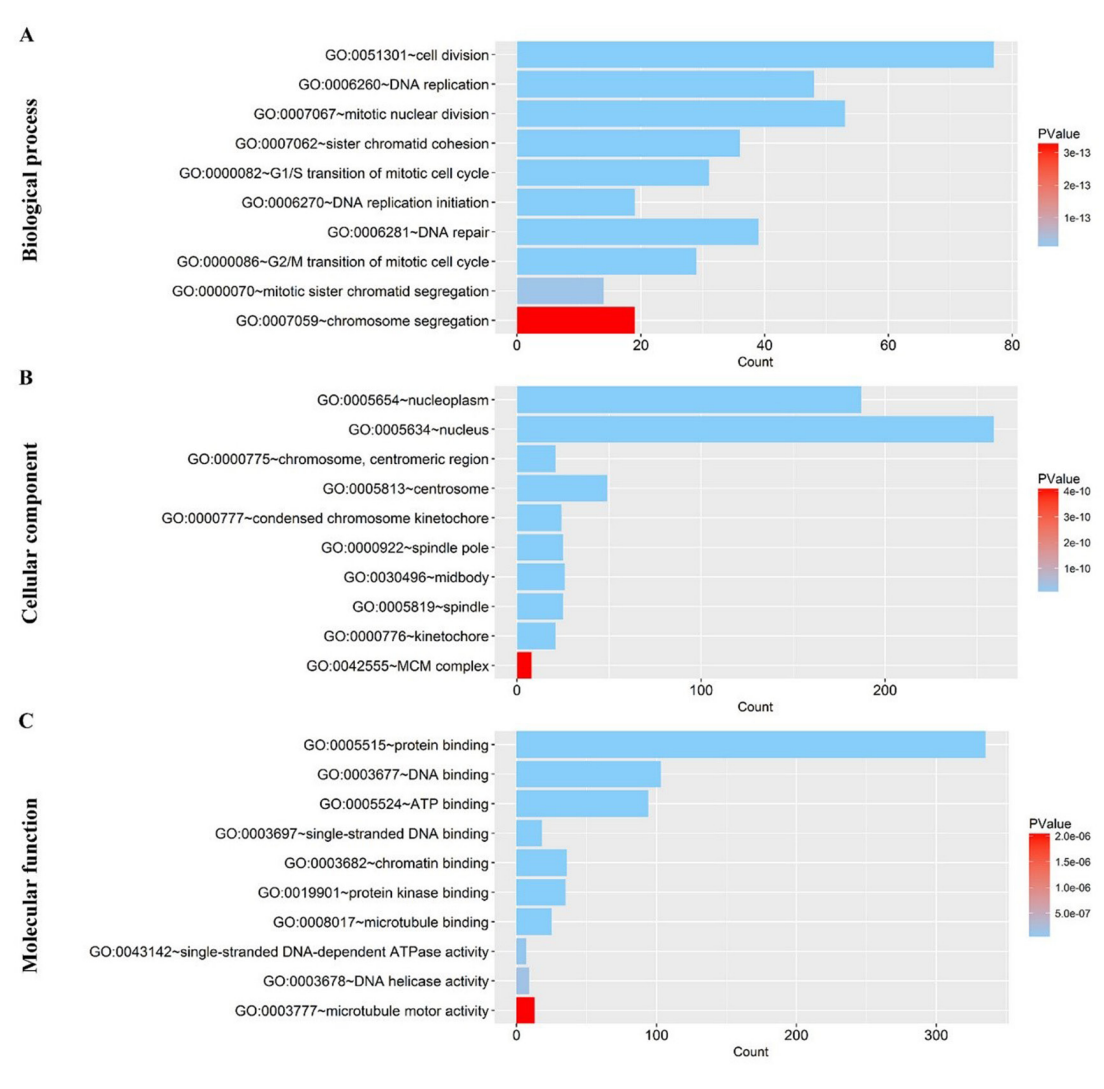
Gene Ontology analysis of the CDK5-related genes in HCC (**A**) Biological process; (**B**) Cellular component; (**C**) Molecular function. The plot was generated using the ggplot2 package of R language.

**Figure 12:**
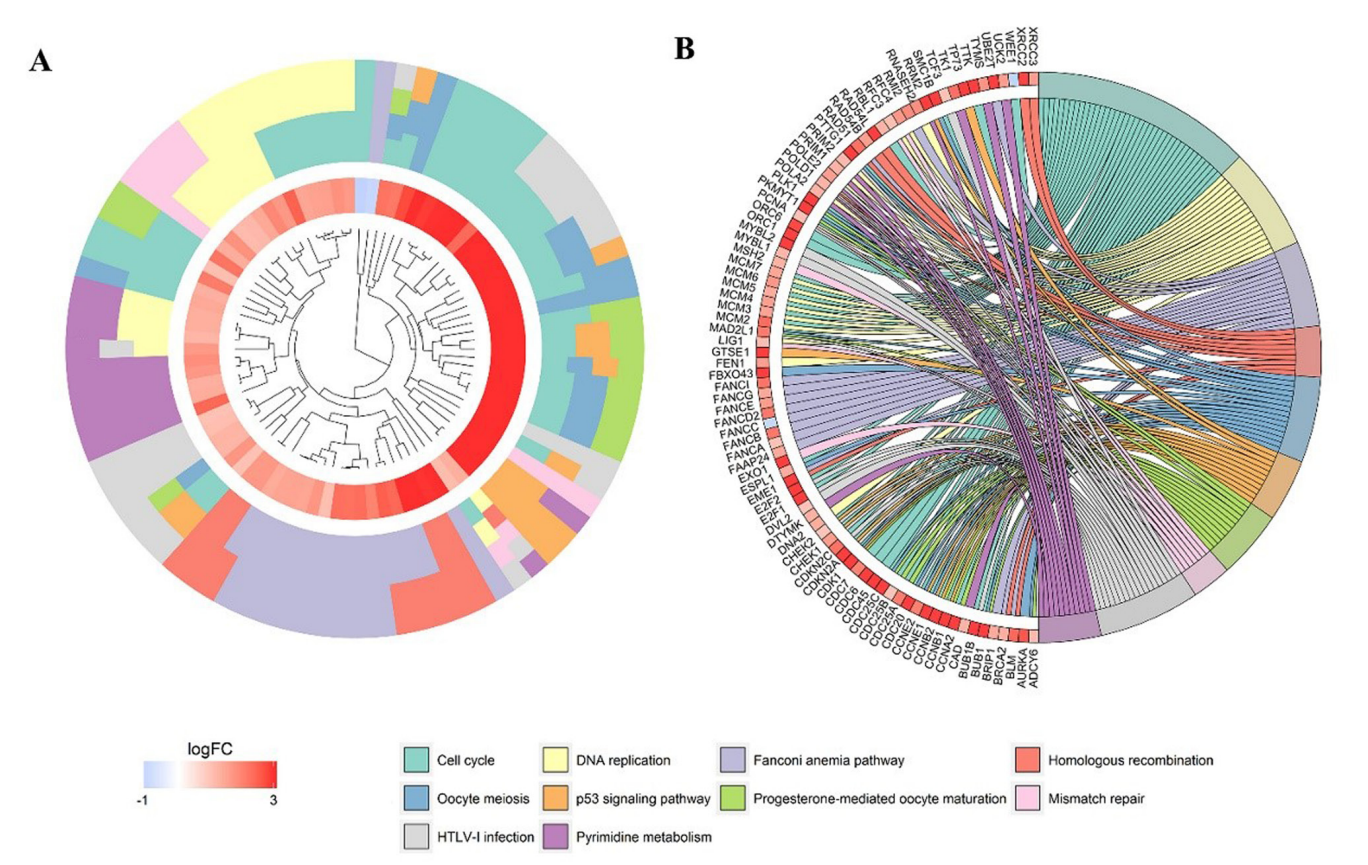
Significantly enriched annotation of the Kyoto Encyclopedia of Genes and Genomes (KEGG) pathway analysis of CDK5-related genes in HCC (**A**) Cluster plot displays a circular dendrogram of the clustering of the expression profiles. The inner ring displays the color-coded logFC, whereas the outer ring indicates the assigned functional KEGG pathways. (**B**) In the Chord plot, related genes are linked to their enriched KEGG pathways via ribbons. Red coding next to the selected genes indicated up-regulation and blue ones indicated down-regulation.

After the calculation described above, 4824 diﬀerently expressed genes (DEGs) were obtained when considering a stringent threshold of |log2FC|>1 and Padj<0.05 (Figure 10A). Then, the Weighted Gene Co-Expression Network Analysis (WGCNA) integrated function was used to calculate a set of genes related to CDK5. As shown in Figure 10B, the visualized heatmap indicated that 542 genes clustered in turquoise were most significant correlated with CDK5 and several clinicopathological parameters. To further investigate the functional associations of CDK5-related genes, we performed GO and KEGG pathway annotation analysis and displayed the top 10 pathways of Oncology (GO) and the Kyoto Encyclopedia of Genes and Genomes (KEGG) in Table 4. As shown in Figure 11, the majority of the CDK5-relevant genes were significantly represented by the GO biological categories of “cell division”, “DNA replication” and “mitotic nuclear division”. Regarding the cellular component, “nucleoplasm”, “nucleus” and “chromosome, centromeric region” represent the three most significantly enriched terms. Regarding molecular function, the genes were markedly represented by “protein binding”, “DNA binding” and “ATP binding”. KEGG pathway analysis revealed that “Cell cycle” was the most significant pathway related with CDK5-related genes (Figure 12).

**Table 4:** Top 10 significant pathways of GO and KEGG terms

Category	ID	Term	Counts	Genes	*P*-Value
Biological process	GO:0051301	cell division	77	KIFC1, STOX1, BORA, KNTC1, CUZD1, AURKA, PTTG1, FAM83D, CCNE2, KIF2C etc.	8.38E-46
Biological process	GO:0006260	DNA replication	48	CLSPN, BLM, TICRR, KIAA0101, CHEK1, POLA2, MCM10, CDT1, CDC45, MCM8 etc.	1.30E-35
Biological process	GO:0007067	mitotic nuclear division	53	STOX1, BORA, KNTC1, PKMYT1, AURKA, AURKB, PTTG1, FAM83D, KIF2C, OIP5 etc.	1.33E-30
Biological process	GO:0007062	sister chromatid cohesion	36	KNTC1, AURKB, SPC24, SPC25, KIF2C, CDCA8, DDX11, CENPA, INCENP, BUB1 etc.	1.08E-28
Biological process	GO:0000082	G1/S transition of mitotic cell	31	IQGAP3, PKMYT1, POLA2, MCM10, CDT1, CCNE2, PRIM1, CCNE1, TYMS, CDC45 etc.	1.14E-22
Biological process	GO:0006270	DNA replication initiation	19	CDC7, CDC6, GINS4, POLA2, MCM2, MCM10, MCM3, MCM4, MCM5, MCM6 etc.	3.02E-20
Biological process	GO:0006281	DNA repair	39	CLSPN, XRCC3, XRCC2, BLM, TICRR, FOXM1, FAAP24, CHEK1, PTTG1, ANKLE1 etc.	1.66E-18
Biological process	GO:0000086	G2/M transition of mitotic cell	29	CEP72, HAUS5, NEK2, FOXM1, BORA, PKMYT1, CHEK1, AURKA, CHEK2, HMMR etc.	1.13E-16
Biological process	GO:0000070	mitotic sister chromatid segreg	14	KIFC1, NEK2, DSN1, NUSAP1, KIF18B, ESPL1, NDC80, KNSTRN, SMC4, MAD2L1 etc.	2.34E-14
Biological process	GO:0007059	chromosome segregation	19	KIF11, NEK2, DSN1, NUF2, CENPF, NDC80, CENPE, KNSTRN, ESCO2, SPC25 etc.	3.26E-13
Cellular component	GO:0005654	nucleoplasm	187	XRCC3, DBF4B, XRCC2, PRC1, NR2C2AP, PKMYT1, CBX2, AURKA, AURKB, MCM10 etc.	7.93E-34
Cellular component	GO:0005634	nucleus	259	KIFC1, XRCC3, DBF4B, RUSC1, PRR11, AURKA, AURKB, PTTG1, ANKLE1, MAMSTR etc.	7.57E-25
Cellular component	GO:0000775	chromosome, centromeric region	21	DNMT3A, CENPL, MKI67, CENPQ, CENPP, NUF2, CENPF, NDC80, BIRC5, CENPE etc.	2.11E-17
Cellular component	GO:0005813	centrosome	49	KIF23, STIL, CEP72, STOX1, HAUS5, XRCC2, NEK2, AURKA, CHEK1, CEP55 etc.	8.62E-17
Cellular component	GO:0000777	condensed chromosome kinetochor	24	CENPO, CENPM, NEK2, NUF2, KNTC1, BIRC5, NDC80, CENPE, KNSTRN, CENPK etc.	1.02E-16
Cellular component	GO:0000922	spindle pole	25	PRC1, NEK2, KNTC1, FBF1, DDX11, GPSM2, CKAP2, CDC6, KIF11, DSN1 etc.	2.20E-15
Cellular component	GO:0030496	midbody	26	KIF23, KIF4A, PRC1, NEK2, AURKA, AURKB, CEP55, CDCA8, DDX11, INCENP etc.	1.33E-14
Cellular component	GO:0005819	spindle	25	KIF23, KIFC1, HAUS5, PRC1, TTK, AURKA, AURKB, ATAT1, SAC3D1, INCENP etc.	2.63E-14
Cellular component	GO:0000776	kinetochore	21	NEK2, KIF18A, TTK, CENPF, NDC80, CENPE, AURKB, KNSTRN, CENPI, CENPH etc.	4.64E-14
Cellular component	GO:0042555	MCM complex	8	MCM7, MMS22L, TONSL, MCM2, MCM3, MCM4, MCM5, MCM6	4.08E-10
Molecular function	GO:0005515	protein binding	335	XRCC3, XRCC2, DBF4B, RUSC1, ADCY6, NR2C2AP, AURKA, AURKB, PTTG1, ANKLE1 etc.	6.94E-18
Molecular function	GO:0003677	DNA binding	103	XRCC3, CBX2, CDKN2A, DDX11, ZNF300, WDR76, PRIM2, TIGD3, ORC6, H2AFX etc.	1.33E-14
Molecular function	GO:0005524	ATP binding	94	KIF23, KIFC1, XRCC3, KIF24, XRCC2, FIGNL1, ADCY6, DTYMK, TTLL4, PKMYT1 etc.	7.80E-14
Molecular function	GO:0003697	single-stranded DNA binding	18	XRCC3, HMGB2, XRCC2, RAD51AP1, BLM, MSH2, NEIL3, BRCA2, MCM10, MCM4 etc.	6.70E-10
Molecular function	GO:0003682	chromatin binding	36	TICRR, EZH2, KIAA0101, FAAP24, CBX2, ZKSCAN3, CDC45, DDX11, CENPA, POLQ etc.	1.23E-09
Molecular function	GO:0019901	protein kinase binding	35	E2F1, CKS1B, TRAF2, CDK5R1, DBF4B, PRC1, FOXM1, BORA, ADCY6, AURKA etc.	1.65E-09
Molecular function	GO:0008017	microtubule binding	25	GAS2L3, KIF14, KIF23, KIFC1, ARHGEF2, KIF4A, KIF24, KIF11, PRC1, KIF15 etc.	4.03E-09
Molecular function	GO:0043142	single-stranded DNA-dependent A	7	DNA2, RFC3, RFC4, CHTF18, POLQ, RAD51, DSCC1	8.62E-08
Molecular function	GO:0003678	DNA helicase activity	9	DNA2, MCM7, PIF1, RAD54B, MCM2, MCM3, MCM4, MCM5, MCM6	1.69E-07
Molecular function	GO:0003777	microtubule motor activity	13	KIF14, KIF23, KIFC1, KIF4A, KIF24, KIF11, KIF15, KIF18A, KIF18B, CENPE etc.	2.04E-06
KEGG_PATHWAY	hsa04110	Cell cycle	39	E2F1, E2F2, PKMYT1, TTK, CHEK1, PTTG1, CHEK2, CCNE2, CCNE1, CDC45 etc.	2.82E-30
KEGG_PATHWAY	hsa03030	DNA replication	18	LIG1, POLA2, MCM2, RNASEH2A, MCM3, MCM4, MCM5, MCM6, PRIM1, DNA2 etc.	8.95E-17
KEGG_PATHWAY	hsa03460	Fanconi anemia pathway	15	BLM, EME1, FAAP24, BRCA2, BRIP1, RMI2, RAD51, FANCI, FANCD2, FANCE etc.	1.00E-09
KEGG_PATHWAY	hsa03440	Homologous recombination	9	XRCC3, XRCC2, BLM, POLD1, EME1, BRCA2, RAD54B, RAD54L, RAD51	1.34E-05
KEGG_PATHWAY	hsa04114	Oocyte meiosis	15	CDK1, ADCY6, PKMYT1, CDC20, ESPL1, AURKA, PTTG1, CDC25C, CCNE2, CCNE1 etc.	1.22E-05
KEGG_PATHWAY	hsa04115	p53 signaling pathway	11	CCNB1, CCNE2, CCNE1, CDK1, CDKN2A, CCNB2, RRM2, CHEK1, CHEK2, GTSE1 etc.	1.30E-04
KEGG_PATHWAY	hsa04914	Progesterone-mediated oocyte maturation	12	CCNB1, CDK1, MAD2L1, CCNB2, PLK1, ADCY6, BUB1, PKMYT1, CDC25C, CCNA2 etc.	1.94E-04
KEGG_PATHWAY	hsa03430	Mismatch repair	7	EXO1, RFC3, RFC4, MSH2, LIG1, POLD1, PCNA	3.13E-04
KEGG_PATHWAY	hsa05166	HTLV-I infection	18	DVL2, E2F1, E2F2, ADCY6, CHEK1, CDC20, PTTG1, MYBL1, CHEK2, MYBL2 etc.	0.003175
KEGG_PATHWAY	hsa00240	Pyrimidine metabolism	11	PRIM1, TYMS, POLE2, POLD1, RRM2, DTYMK, PRIM2, CAD, UCK2, POLA2 etc.	0.003769

### CDK5-siRNA inhibited cell growth and induced apoptosis *in vitro*

**Figure 13:**
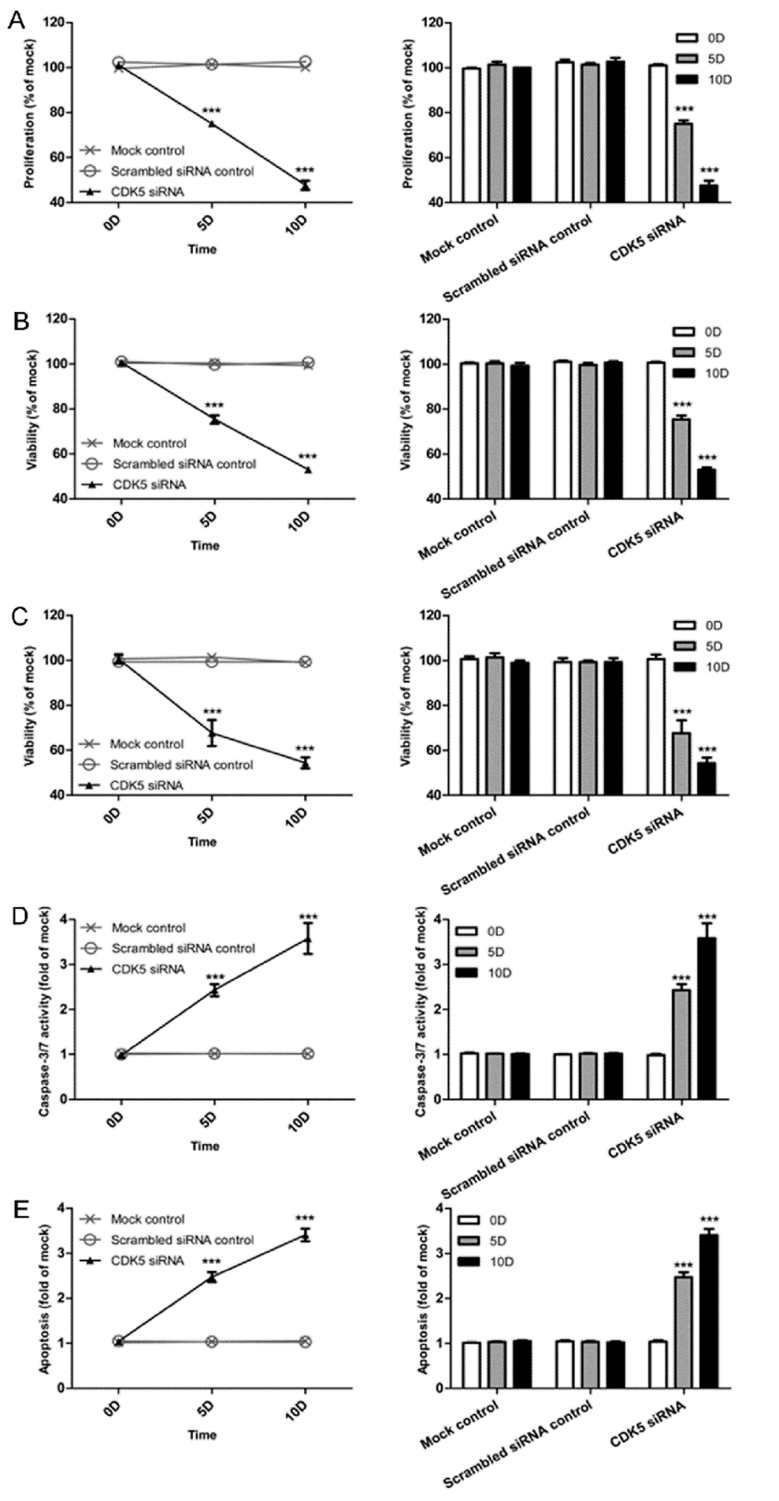
Effects of CDK5-specific-siRNA on cell growth and apoptosis in HCC HepB3 cells (**A**) Cell proliferation detected using an MTS assay. (**B**) Cell viability assessed with a fluorimetric assay. (**C**) Cell viability assessed with Hoechst33342 and PI double fluorescent staining. (**D**) Caspase-3/7 activity. (**E**) Cell apoptosis detected by Hoechst33342 and PI double fluorescent assay. (** *P*<0.01 and *** *P*<0.001 compared with mock control).

**Figure 14:**
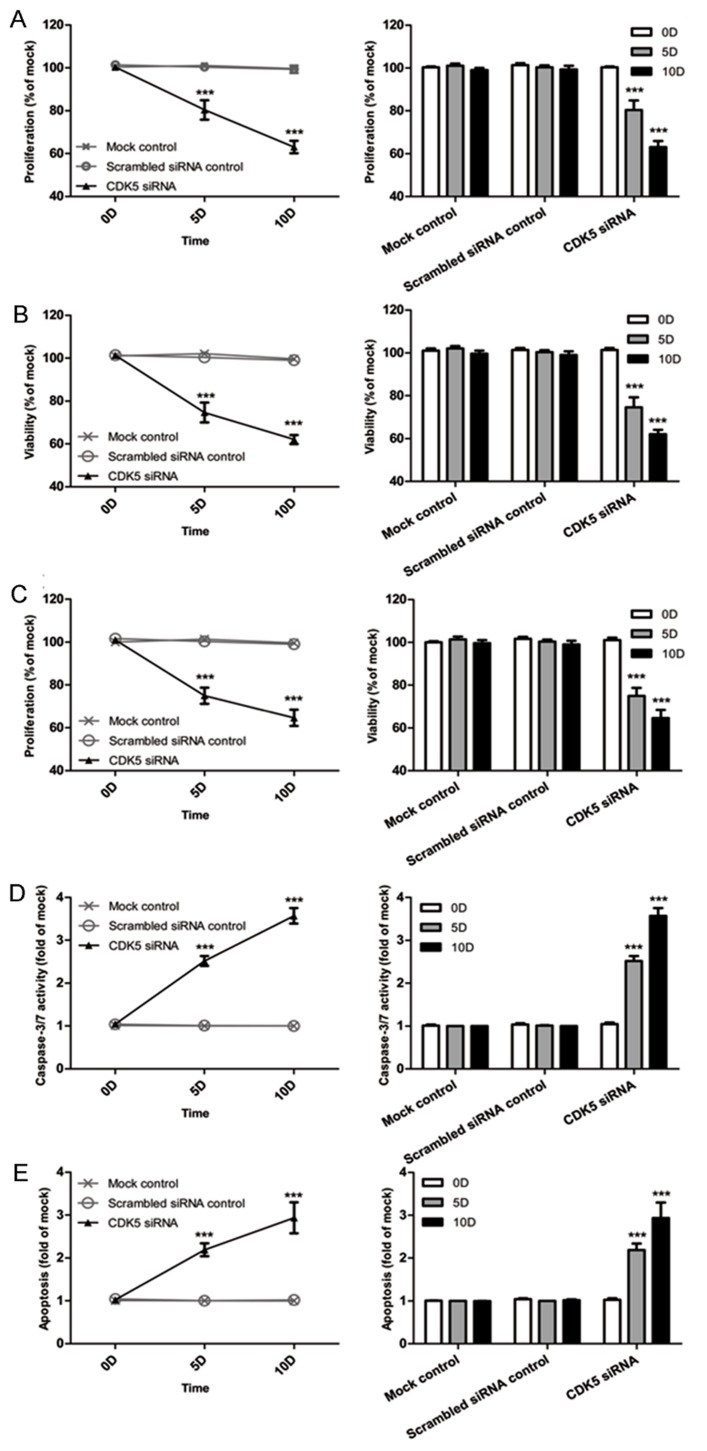
Effects of CDK5-specific-siRNA on cell growth and apoptosis in HCC HepG2 cells (**A**) Cell proliferation detected using an MTS assay. (**B**) Cell viability assessed with a fluorimetric assay. (**C**) Cell viability assessed with Hoechst33342 and PI double fluorescent staining. (**D**) Caspase-3/7 activity. (**E**) Cell apoptosis detected by Hoechst33342 and PI double fluorescent assay. (** *P*<0.01 and *** *P*<0.001 compared with mock control).

**Figure 15:**
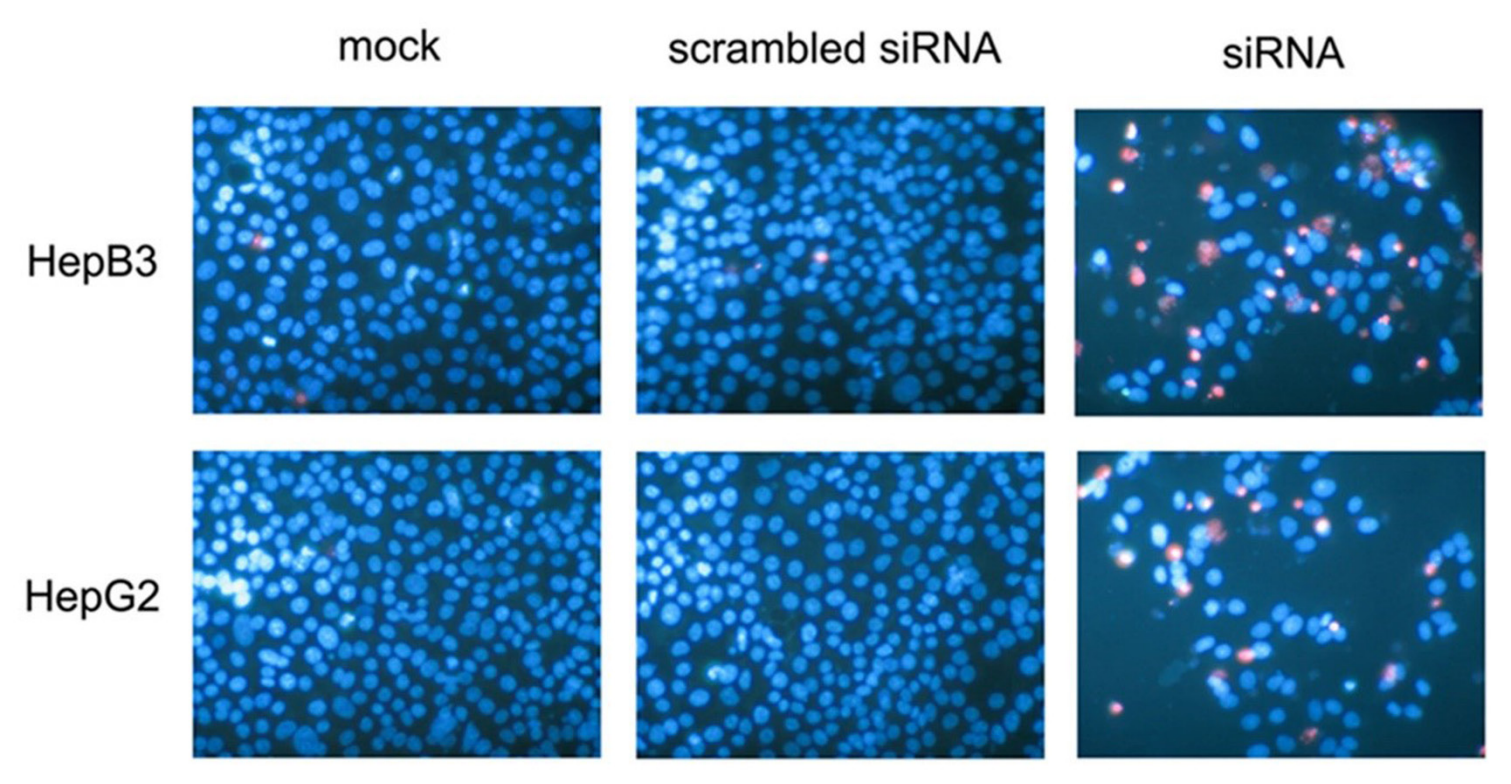
Effects of CDK5-specific-siRNA detected by Hoechst33342 and PI double fluorescent staining HepB3 and HepG2 cell lines were treated with CDK5-specific-siRNA. Live cells and apoptotic cells were detected with Hoechst33342 and PI double fluorescent staining on the 10^th^ day.

A colorimetric MTS tetrazolium assay was performed to detect HepG2 and HepB3 cell growth. A reduction in cell proliferation in the CDK5-siRNA group was noted compared with the mock control in both cell lines (*P*=0.001) (Figure 13A, Figure 14A). HepG2 cell growth was reduced by 20% and 40% at 5 days and 10 days after transfection, respectively, whereas the reduction of HepB3 cell growth even reached 25% and 50% at 5 days and 10 days after transfection, respectively. Moreover, fluorimetric resorufin viability assay and Hoe/PI results largely mirrored the MTS tetrazolium assay results (Figure 13B, Figure 14C). A fluorescent caspase-3/7 assay was adopted in this study, revealing an increase in the caspase-3/7 signal in both HepG2 and HepB3 cells transfected with CDK5-siRNA. Caspase-3/7 activity in the CDK5-siRNA group in both HepG2 and HepB3 cells was approximately 2.5-fold increased compared with control and scrambled siRNA control 10 days after transfection (Figure 13D, Figure 14D). To confirm the results, Hoe/PI assays were performed to measure cell apoptosis based on microscopic counting of apoptotic cells. The results were similar to the fluorescent caspase-3/7 assay results, demonstrating that apoptosis activity in the CDK5-siRNA group was approximately two-fold increased compared with the mock control and scrambled siRNA control in both HepG2 and HepB3 cells (Figure 13E, Figure 14E, Figure 15).

## DISCUSSION

The estimated worldwide incidence of liver cancer is 626,000 cases a year. Greater than 50% of cases are from China. Approximately 745,000 people die from HCC yearly worldwide [20, 21]. HCC represents a large portion of primary liver cancer [22]. However, diagnostic methods are limited to date. In addition, HCC progression is associated with various factors, such as alcoholic cirrhosis, hepatitis virus infection, and non-alcoholic steatohepatitis (NASH) [21, 23]. However, cancer genes, including CDK5 and STAT3, represent the most influential factors [24]. Previous studies revealed that CDK5 activity is induced by non-cyclin proteins, including Cdk5R1 (p35) and Cdk5R2 (p39), but CDK5 does not interact with cyclins directly [25]. In addition, CDK5 was mainly investigated as an important regulatory gene in the central nervous system (CNS) and as a potential cause of Alzheimer’s disease (AD) [26]. Recent research revealed that P35 degradation occurs by both ubiquitin-dependent and ubiquitin-independent pathways. P35 degradation leads to the inhibition of P25 expression, which could over-activate CDK5 to induce neuronal cell death [27]. Various experiments demonstrated the significant role of CDK5 in the CNS by broadly disrupting in neuronal layering of various brain structures, such as the cerebral cortex, cerebellum, hippocampus and olfactory bulb [28]. Thus, in our study, we paid more attention to investigating the relationship between CDK5 and clinicopathological parameters, as well as the diagnostic and prognostic value of CDK5. Here, we collected 412 samples (HCC, n=171; adjacent non-HCC liver tissues, n=171; normal liver tissues, n=33; cirrhotic tissues, n=37) from surgically resected samples. Meanwhile, a total of 2238 HCC tissues and 2045 non-cancerous tissues were deeply mined and integrated from various public datasets. Thus, based on large-scale sample size, the results of CDK5 significantly overexpressed in HCC patients would be more reliable and valuable. Furthermore, our *in vitro* study found that CDK5 could inhibit cell growth and induce apoptosis in HCC cell lines, which might be the mechanism by which CDK5 critically impacted the initiation and development of HCC.

Our study first demonstrated a significant value of CDK5 in the clinical diagnosis of HCC. CDK5 expression is up-regulated in HCC compared with normal tissues based on immunohistochemistry performed in our study. A similar pattern was revealed by the high-throughput RNA-seq analysis. Therefore, CDK5 over-expression is likely associated with the occurrence of HCC, and further studies should be performed regarding the role of CDK5 expression in HCC diagnosis and individualized treatment. Moreover, compared with cirrhotic and para-carcinoma tissues in the liver, CDK5 expression was increased in HCC (*P*<0.001). However, CDK5 expression exhibited no significant differences between cirrhotic and normal tissues in the liver based on immunohistochemistry, indicating that CDK5 is specifically over-expressed in HCC and providing a new marker to distinguish HCC from other hepatic diseases, such as cirrhosis, thus improving the diagnosis accuracy of HCC. Furthermore, from the meta-analysis results of TCGA and other open databases, CDK5 expression in HCC was significantly increased compared with non-HCC liver cancer. Furthermore, ROC analysis was performed in our immunohistochemistry study and revealed that the CDK5 expression level was most useful in the diagnosis of tumor metastasis followed by tissue types, TNM stage, size, embolus and vaso-invasion. These results provide effective target molecules for an accurate diagnosis of HCC compared with other tissue types and to predict HCC progression. Similarly, ROC analysis results of data from the TCGA database confirmed that CDK5 could play an effective role in distinguishing HCC from normal tissues.

In addition, CDK5 may be an effective biomarker for HCC staging. Our immunohistochemistry results revealed increased CDK5 expression levels in HCC patients with tumor metastasis, vascular invasion, portal vein tumor embolus, moderate differentiation and higher clinical TNM stages. Greater than 97.5% of HCC patients with metastasis exhibited increased CDK5 expression. Therefore, we can easily infer that increased CDK5 expression levels are related to more advanced stages of HCC. These results suggest that the CDK5 expression level detection may represent a good choice to distinguish the stage of HCC. Based on the analysis of data extracted from the TCGA database, CDK5 expression correlated with patient age. Nevertheless, no significant correlation was noted between CDK5 and other clinical parameters, such as pathologic stage, and HCC histological grade and race, revealing a different trend compared with our immunohistochemistry results. All our immunohistochemistry samples were obtained from Chinese individuals, whereas cases from the TCGA database were obtained from various populations worldwide. This difference may explain the different results obtained from our immunohistochemistry analysis and the high-through RNA-seq analysis.

However, whether CDK5 represents a suitable biomarker for the prediction of HCC prognosis remains controversial. The survival analysis based on the TCGA database revealed that the CDK5 expression levels were significantly related to both overall survival (OS) and disease-free survival (DFS). Thus, CDK5 may effectively predict HCC prognosis. However, our RNA-seq results demonstrated that CDK5 could act as a statistically effective HCC prognostic biomarker. Given that the algorithm used in these methods differed, the clinic value of CDK5 in HCC prognosis requires further investigation. In addition to its clinical value, the mechanism by which CDK5 regulates the initiation and development of HCC requires further study.

Based on bioinformatics methods, we hypothesize that CDK5 exercises its functions via several proliferative signaling pathways. We confirmed our hypothesis in a series of *in vitro* experiments. In our *in vitro* experiment, cell proliferation was inhibited in the CDK5-siRNA group, suggesting that CDK5 promotes cell proliferation and subsequently triggers HCC progression. In addition, HCC cell apoptosis increased when CDK5 expression was suppressed, indicating that CDK5 down-regulation induces the low apoptosis rates. Of note, three different methods were adopted to detect the proliferation of both HCC cell lines in our *in vitro* study, revealing the same trend of cell proliferation. In addition, two different methods were performed to measure apoptosis in both HCC cell lines, revealing a similar trend in cell apoptosis. Thus, the results of our *in vitro* experiment are reliable. Similarly, Liu JL. Et al.’s study on CDK5 and lung cancer revealed a similar CDK5 proliferation and apoptosis trend in lung cancer cell lines when CDK5 activity was suppressed by siRNA [29]. A paradoxical mechanism of CDK5 in HCC was previously reported. Most recently, CDK5 was reported to promote angiogenesis in HCC [16]. As demonstrated by previous CDK5 studies, CDK5 interacts with numerous types of proteins, such as β-catenin, GFAP, and α-actinin [30]. CDK5 activity is dependent on p35/p39 binding. CDK5 and p35 were recently identified as a potent tumor suppressor in HCC. The decreased expression of p39 correlated with a poor overall survival rate [31]. Regulation of CDK5 activity promoted the proliferation of medullary thyroid carcinoma (MTC) [32]. In other studies, CDK5 promoted medullary thyroid carcinoma cell growth by regulating STAT3 activation and cell proliferation [24]. Feldmann et.al concluded that inhibiting CDK5 could suppress Ras-Ral signaling, blocking pancreatic cancer formation and progression [33]. In addition, emerging evidence indicates that CDK5 functions in prostate cancer cells through the control of cell-motility and metastatic potential [34]. Sustaining proliferative signaling has been recognized as a fundamental hallmark of cancers. Cell growth disturbances implicated in the regulation of the progression and migration of cancer cell arguably [35]. Based on bioinformatics methods, we found that co-expressed genes of CDK5 enriched in several pro-proliferative pathways, such as cell cycle and DNA replication. Therefore, we hypothesize that CDK5 exercises its functions in tumorigenesis and progression via disturbing cell growth and apoptosis. Taken together, these findings indicated that CDK5 is involved in numerous steps during cancer progression.

In summary, CDK5 plays an essential role in HCC initiation and progression, most likely via accelerating proliferation and suppressing apoptosis in HCC cells.

## MATERIALS AND METHODS

### Immunohistochemical technique

In the present study, 412 surgically resected tissue samples were obtained from the First Affiliated Hospital of Guangxi Medical University (Nanning, Guangxi, China). The 412 tissues included 33 normal liver tissues, 37 cirrhosis tissues, 171 adjacent non-HCC liver tissues and 171 primary HCC tissues. HCC was diagnosed according to WHO classification of tumors of the digestive system (http://www.who.int/en/). The age of all the patients ranged from 28 to 76 years (mean, 51 years). All clinicopathological information was obtained from medical records and summarized in Table 1. The protocol of our study was approved by the Ethical Committee of the First Affiliated Hospital of Guangxi Medical University. Patients and clinicians provided written informed consent permitting the use the samples. All the samples were diagnosed and reviewed by two independent pathologists.

Immunohistochemistry was applied to measure the CDK5 expression level of the samples. Regarding quantification of CDK5 immunopositive staining, the positive cells exhibit yellow to brown color in the nucleus and/or cytoplasm. A total of one hundred cells were evaluated from 10 representative regions from each case. The immunohistochemistry results were analyzed according to staining intensity, immunodetection and the number of positive cells. We evaluated the results of staining individually to achieve a final agreement regarding controversial cases using a multihead microscope.

Based on the following criteria, CDK5 expression was classified semiquantitatively as follows: no staining was recorded as 0; weak staining with focal or fine granular morphology was recorded as 1; linear or cluster, strong staining was recorded as 2; and diffuse, intense staining was recorded as 3. The score ranged from 0 to 3 for the percentage of positive cells in each scenario. A score of 0 was recorded when no staining was observed. A score of 1 indicated that less than 30% of cells were stained. A score of 2 indicated that 30% to 70% of cells were positive. If greater than 70% cells were positive, a score of 3 was recorded. The samples were then categorized as positive or negative based on the sum of the scores as follows: score 0–2 implied negative; 3 implied weakly positive (+); 4 implied moderately positive (++) and 5–6 implied strongly positive (+++). Any score greater than 3 in the present study was considered to indicate positive expression in this study.

### TCGA dataset

CDK5 expression was analyzed by file data downloaded from the TCGA database (http://cancergenome.nih.gov/). The CDK5 expression data consist of individual 374 HCC samples and 50 normal controls. Clinicopathological parameters, including age, gender, tumor status, race, relative family cancer history, histological grade, TNM stage, pathological T stage, pathological N stage, pathological M stage and vascular tumor cell type, were also estimated. The data above were used to assess the correlation between CDK5 levels and prognosis as presented in the results.

### Other open databases

To further examine the CDK5 expression pattern in HCC, we collected HCC-relevant RNA-seq and microarray datasets from GEO (https://www.ncbi.nlm.nih.gov/geo/), ArrayExpress (http://www.ebi.ac.uk/arrayexpress/), and Oncomine (https://www.oncomine.org/resource/login.html) databases. The following search words were employed: (malignan* OR cancer OR tumour OR tumour OR neoplas* OR carcinoma) AND (hepatocellular OR liver OR hepatic OR HCC).

### Bioinformatic analysis

The RNA-Seq data of liver HCC downloaded from TCGA were analyzed using the Limma package of R language (http://www.bioconductor.org/packages/release/bioc/html/limma.html) to identify DEGs between liver HCC and non-tumor tissues. DEGs were selected based on the following criteria: Padj<0.05 and |log2 Fold Change| (|log2FC|) >1. Next, WGCNA, an algorithm for the identification of co-expression gene modules, was performed to compute a set of genes related to CDK5. The process was accomplished using the WGCNA package of the R language (https://labs.genetics.ucla.edu/horvath/CoexpressionNetwork/Rpackages/WGCNA/). For CDK5-related genes, GO and KEGG pathway analyses were performed by the online bioinformatic tool The Database for Annotation, Visualization and Integrated Discovery (DAVID) v6.8 and visualized by the R package ‘GOplot’ and ‘ggplot2’.

### Experiment *in vitro*

The human hepatic cell lines HepG2 and HepB3 were purchased from the American Type Culture Collection (ATCC, Rockville, MD, USA). CDK5-siRNA was obtained from Sangon Biotech (Shanghai, China) [17].

### Viability

Cell viability was measured by fluorimetric detection of resorufin. The procedure was performed per the manufacturer’s instructions. After transfecting CDK5-siRNA in HepG2 and HepB3 cell lines, cell viability was assayed at 0, 5, and 10 days and compared with mock controls and scrambled siRNA controls.

### Cell proliferation

To further verify the cell viability assay data obtained as described above, cell proliferation was measured using a colorimetric tetrazolium (MTS) assay.

### Caspase-3/7 activity detection

A synthetic rhodamine-labeled caspase-3/7 substrate (Apo-ONEW Homogeneous caspase-3/7 Assay, G7790, Promega, Madison, WI, USA) was used to measure caspase-3/7 activity immediately after the detection of the cell viability as described above. The procedure was performed per the instructions of the manufacturer.

### Evaluation of cell apoptosis and morphology using fluorescence microscopy

The impact of CDK5 siRNAs on apoptosis in cell lines was assayed using Hoechst 33342 and propidium iodide (PI) double fluorescent chromatin staining as described in our previous study [17-19]. Briefly, HepG2 and HepB3 cells were treated with Hoechst 33342 (5 µg/ml) after centrifugation at 1500 rpm. Then, cells were stained with PI for 15 min in the dark. The apoptotic rate was obtained from the comparison of the number of apoptotic cells from distinct experimental groups/the number of viable cells in the same well.

### Statistical analysis

SPSS 22.0 (SPSS Inc., Chicago, IL, USA) was applied for statistical analysis of IHC results. Pearson Chi-Square tests was used to evaluate the significance of the role CDK5 in the HCC pathological categories. Pearson Chi-Square tests were also performed to compare CDK5 expression based on the parameters of age, gender, tumor stage (TNM), lymph node metastasis and distal metastasis. The associations between CDK5 expression levels and the clinicopathological characteristics were evaluated using Spearman’s correlation. The diagnostic value of CDK5 was identified by employing ROC. P-values less than 0.05 indicated a statistically significant difference.

Regarding data from the TCGA and other public databases, SPSS 22.0 was also used for statistical analysis. R, OriginPro 2017 (Northampton, Massachusetts, USA), and GraphPad Prism 5 (San Diego, CA, USA) were used to plot figures. Data were presented as mean±SD in each of the datasets. The independent-samples T test was used to compare the differential CDK5 expression level in different patients (HCC vs. Normal). Similarly, CDK5 expression level in clinicopathological parameters, such as tumor stage (TNM), age, gender, histological stage, and race, were analyzed by independent-samples T test separately. ROC was employed to identify the diagnostic value of CDK5 protein in HCC. Statistical significance was determined at *P*<0.05.

To obtain a comprehensive perspective on CDK5 expression, we integrated multiple source data in the form of meta-analysis using STATA 12.0 (StataCorp, College Station, TX, USA). The total SMD was computed. When SMD>0 and its 95% CI did not cross, an integer of 0 indicated that CDK5 in tumors is significantly overexpressed compared with adjacent non-tumor tissues. To further study the comprehensive efficiency of CDK5 in distinguishing tumor from non-tumor tissues, we generated SROC curves and calculated the AUC value with 95% CI, sensitivity and specificity.

*In vitro* experimental data were analyzed by SPSS and graphed using GraphPad Prism 5 (GraphPad Software Inc., La Jolla, CA, USA) directly. Appropriate graphs (category graph, symbols and lines, interleaved bars, and vertical) were generated to represent the relationship between CDK5 and proliferation as well as HCC cell apoptosis.
